# Penetration of Different Impression Materials into Exposed Dentinal Tubules during the Impression Procedure

**DOI:** 10.3390/ma13061321

**Published:** 2020-03-14

**Authors:** Bruna Sinjari, Gianmaria D’Addazio, Edit Xhajanka, Sergio Caputi, Giuseppe Varvara, Tonino Traini

**Affiliations:** 1 Department of Medical Oral and Biotechnological Sciences, University “G. d’Annunzio” of Chieti-Pescara, Via dei Vestini 31, 66100 Chieti (CH), Italy; gianmariad@gmail.com (G.D.); scaputi@unich.it (S.C.); t.traini@unich.it (T.T.); 2 Department of Dental Medicine, Medical University of Tirana, Rruga e Dibrës, Tirana 1001, Albania; editxhajanka@yahoo.com

**Keywords:** immediate dentine sealing, impression material, adhesive dentistry, prosthetic dentistry, in vitro

## Abstract

Adhesive restorations have been shown to guarantee excellent performance and longevity, although this comes with some disadvantages. Among these, the vulnerability of dentine to different agents has been widely evaluated. The aim of this study was to evaluate the possible penetration of impression materials into freshly cut dentine. Dentine from 27 teeth was impressed with polyether (Impregum Penta L) (nine teeth) and with polyvinyl siloxane (Aquasil Ultra LV) (nine teeth). The surface of nine teeth after the impressions were used as the control. Specifically, the extroflections caused by the imprinting of the dentinal tubules on the impression material, the so-called impression tags, were measured. Furthermore, the presence of the material inside the tubules was examined. Scanning electron microscopy analysis showed material tags for all of the experimental groups. The mean lengths (±SD) were 22.6 (±11.0) µm for polyether, 21.8 (±12.8) µm for polyvinyl siloxane and 11.3 (±7.0) µm for the tooth control, with mean diameters (±SD) of 2.8 (±0.5), 2.4 (±0.7) and 3.1 (±0.7) µm, respectively. Fractal analysis showed fractal dimensions of 1.78 (±0.03), 1.77 (±0.03) and 1.71 (±0.03), respectively. These data demonstrated that the impression materials can remain inside the dentinal tubules, which can adversely affect the adhesive procedures.

## 1. Introduction

Studies of dental materials and their interactions with the dental substrate and periodontal tissue have led the availability of increasingly high-performance materials and specific protocols for their use [1]. Since their introduction, adhesive restoration materials, such as composite resins and glass ceramics, have increased in popularity due to their optical properties and the reduced need for tissue removal [2].

Specifically, the introduction of adhesive dentistry has made it possible to create restorations with high retention and low potential for detachment, accompanied by high aesthetic characteristics and reduced invasiveness [3]. For these reasons, composite resin is now the first-choice material to restore anterior and posterior teeth during direct restorations [4,5]. Among these composite resins, different materials have been developed. Indeed, after the surface treatment of feldspathic porcelain was described in 1983 [6], lithium disilicate glass ceramic has become the most popular material for all ceramic restorations [7].

It is known that the long-term success rate of these materials is influenced by various factors. The most influential of these for effective adhesive restorations include the adhesive procedures, the quality of the remaining tooth and the type of material used [8]. This has led to a growing interest in the study of ceramic materials and their related aspects.

It is clear that each technique and each material will have advantages and disadvantages relating to the protocol used or to the material itself [7,8,9]. The disadvantages of adhesive restorations are linked to their vulnerability to gingival fluid, saliva and blood, which can lead to bacterial leakage, postoperative sensitivity, recurrent caries, tooth discoloration and restoration failure [10,11]. To ensure good function and mechanical resistance of adhesive materials, one of the most important aspects relates to their cementation [8]. Several techniques have been proposed to reduce the risk of failure due to cementation [8,12]. Among the first luting agents used, water-based cements were developed, such as zinc phosphate and glass ionomer cements, although these kinds of luting agents had no adhesion characteristics [12]. Subsequently, resin cements were developed, with properties that included good solubility and adhesion, the need for light curing and whether they are adhesive, self-etching, or self-adhesive [13].

Nevertheless, these luting agents need some kind of conditioning procedure of the enamel and dentine before use. Indeed, different techniques have been developed worldwide to ensure long-term follow-up of these materials [14,15,16,17]. Among these, there are the immediate dentine sealing (IDS) protocols that have been suggested for use before the impression [17]. A technique was proposed in the early 1990s [16], which involved the application of the adhesive directly after the preparation of the tooth, and before taking the impression. In this way, it was possible to seal the dentine tubules with the adhesive resin just after tooth preparation. Many studies have shown that this technique is effective for the protection of the pulpodentine complex, to reduce sensitivity and to improve the bond strength of restorations [18,19]. On the contrary, doubts have been raised about the interactions of the impression materials with the adhesive layer, and the subsequent formation of an oxygen-inhibition layer [18]. An oxygen-inhibition layer will result in a reduced conversion rate of the superficial layer of the resin. This happens because the oxygen can inhibit the radicals involved in the polymerization process [20,21].

Some studies have shown how this negative interaction can be reduced [18,22,23], such as with the use of glycerin jelly during the final light-curing polymerization (the air blocking protocol) [24]. Furthermore, a recent study showed that a good surface cleaning protocol can even reset the interaction with the impression material, to ensure its complete polymerization [23]. Despite these efforts, many clinicians do not use IDS, and instead prefer to use a traditional adhesion protocol just before the cementing. This is probably because there have been only a few clinical studies that have analyzed the adhesion performance using the IDS protocol [8]. In these cases, taking the impression directly after the tooth preparation might lead to some known effects, such as an increase in dentine permeability [25].

Crown preparation of a molar tooth can expose up to two million dentinal tubules, which have diameters from 0.6 to 2.0 µm [25,26]. Here, a smear layer of debris can form on the instrumented surface and pack into the superficial portion of the dentinal tubules, which will block them [27]. However, the risk of pulpal damage after preparation remains, and has been related to various factors, such as invasion by oral bacteria, blood, saliva, provisional cements, handpiece oil and haemostatic liquids [28,29,30]. These contaminating agents and factors can reduce the bond strength of adhesive systems that are commonly used for the final seating of glass-based all-ceramic restorations [31]. Interactions between the adhesive layer and the impression materials have been demonstrated after immediate dentine sealing; however, to the best of our knowledge, there have not been any previous investigations into the effects of impression procedures with elastomeric materials on the freshly cut dentine surface.

The aim of the present study was to evaluate the potential interactions between exposed dentine and different elastomeric impression materials during the impression procedures. Impressions were taken just after tooth preparation and without any treatments. The null hypothesis (H0) under the test considered that there was no penetration and interactions between the dentinal tubules and the impression materials.

## 2. Materials and Methods

### 2.1. Study Sample

Twenty-seven teeth were extracted in the Dental Clinic of the Department of Medical, Oral and Biotechnological Sciences, University of Chieti–Pescara, Chieti, Italy. All of the teeth were free of filling materials.

The patients had the teeth removed for periodontal reasons or following fracture. All subjects gave their informed consent for inclusion before they participated in the study. The study was conducted in accordance with the Declaration of Helsinki, and the protocol was approved by the Inter Institutional Ethics Committee of the ‘G. d’Annunzio’ University of Chieti–Pescara on 21 March 2019 with project number 6. After the extractions, the patients were informed that some of their retrieved teeth would be used for an in vitro study. The extracted teeth were randomly divided into three groups for use in the present study, and they comprised 16 molars, 7 premolars and 5 incisors. All the patients were clinically healthy and without any acute general disease to enable their participation in the abovementioned study. The mean age of the patient to whom teeth were selected for this in vitro study was 56.5 ± 9.9 years old.

### 2.2. Sample Preparation

The occlusal dentine of both the molars and premolars or the buccal dentine of the incisors was exposed using a diamond bur (medium grit) on a hand drill with water spray, at 30,000 revolutions/min (25 CHC; KaVo Dental GmbH, Biberach, Germany), to ensure consistent grinding speed and torque. To reproduce the best standard clinical procedures, all of the specimens were treated by the same clinician (TT). After preparation, the teeth surfaces were cleaned with a water spray for 30 s, and lightly dried with air at room temperature.

**Figure 1:**
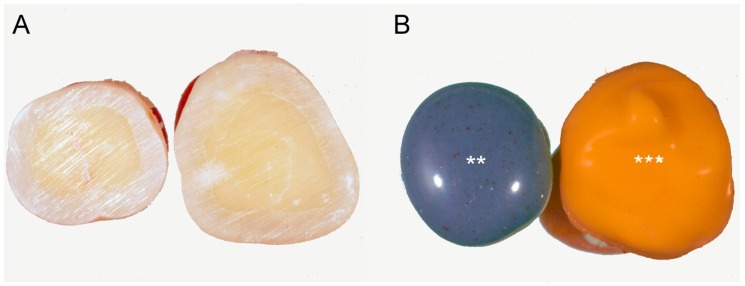
Sample preparation. (**A**) The occlusal dentine was exposed using a diamond bur. (**B**) Impressions taken just after tooth preparation with polyether (**) and polyvinyl siloxane (***).

The prepared surfaces of the specimens were injected with the impression materials, which were subsequently lightly distributed over the impression surfaces using gentle air pressure. The material was left to harden normally as in the clinical setting, following the manufacturer’s instructions, as shown in Figure 1 [32]. In all, nine impressions of randomly chosen teeth were made for each of the experimental groups: polyether (Impregum Penta L; 3M ESPE, Seefeld, Germany); polyvinyl siloxane (PVS; Aquasil Ultra XLV; Dentsply Intl, York, PA, USA) and the surface of the teeth, which was used as the control, with investigation of the exposed surfaces after the impressions.

The specimens were carefully separated from their corresponding teeth and processed for scanning electron microscopy (SEM) analysis (Evo 50; Carl Zeiss, Oberkochen, Germany) of their surface morphology, according to a previously described procedure [33,34]. The samples were sputter coated with gold (K 550; Emitech Ltd., Ashford, Kent, UK) and stored in a sample holder. The SEM set-up was equipped with a tetra solid-state back-scattered electron detector, and was operated at 30 kV accelerating voltage, 10 mm working distance and 870 pA probe current. The images were captured with 20 scans using a line-average technique.

These images were stored as TIFF files before their conversion using Image-Pro Plus version 6.0 (Media Cybernetics Inc., Bethesda, MD, USA), according to their grey levels, which were subsequently binarised. The threshold was adapted to the distribution of the grey values and calibrated for the luminance of the impression material tags. To measure the very complex microshapes of the impressions and the tooth surfaces, both Euclidean geometry, which does not easily lend itself to this goal, and fractal analysis, which has the advantage of being scale invariant, were used.

### 2.3. Variables Measured

The Euclidean analysis measurements were performed on the SEM images to determine the lengths and diameters of the impression material tags. Each image was analyzed using the ImageJ software (ImageJ 1.49 m; Wayne Rasband, National Institute of Health, USA). These measurements were taken for 37 areas for the tooth (control) group, 65 for the PVS group and 58 for the polyether group. The areas analyzed were randomly chosen in each image.

Fractal analysis was used as the shape index, which is defined as a measure of irregularly shaped objects [35]. The microsurface of an impression was considered independent over both the image magnification and resolution (within a given range). Fractal analysis was used to compare the outline of an object for each scale of measurement. This was achieved using a square grid placed over the images. Briefly, grids of various lengths (ε) were used to cover the entire image, from 1 pixel to 45% of the image area, and the number of boxes, N (ε), was recorded for each grid size. The fractal dimension (*D_f_*) was obtained from the negative of the slope of a double-logarithmic plot between N(ε) and ε. Stated mathematically, *D_f_* was estimated using Equation (1):

(1) $$D_{f}=\underset{\varepsilon \to 0}{\lim}[\frac{\ln N\left(\varepsilon \right)}{\ln(\frac{1}{\varepsilon })}]$$

which can be transformed into the power law relationship shown in Equation (2):

(2) $$N{\left(\varepsilon \right)}^{D_{f}}=1$$

where the exponent *D_f_* is an index of the complexity of the surface and also an indicator of how space filling a structure is. The ImageJ plugin known as FracLac (2.5 release 1b5j; A. Karperien, Charles Sturt University, Australia) was used to evaluate the best *D_f_* for all of the models [36], with *D_f_* calculated using the box-counting method [37].

### 2.4. Statistical Analysis

Statistical analysis was performed using the SigmaStat 3.5 statistical package (SPSS Inc., Ekrath, Germany). The results were presented as means and standard deviation. In order to test the normality of the distribution, the Kolmogorov–Smirnov test was used. One-way ANOVA and Tukey’s multiple comparison procedures were used to evaluate differences between the experimental groups. A p value of <0.05 was considered as statistically significant. An alternative Bayesian analysis of variance was used to supplement the comparison of the experimental groups.

## 3. Results

**Figure 2:**
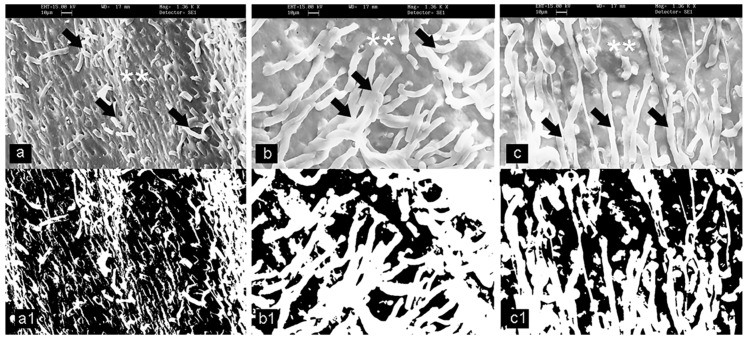
Representative scanning electron microscopy images (**a**–**c**) and corresponding binarized images (**a1**–**c1**) for calculation of the fractal dimension (*D_f_*). (**a**,**a1**) Tooth surface. (**b**,**b1**) Polyvinyl siloxane impression material surface and impression tags as indicated by the black arrows. (**c**,**c1**) Polyether impression material surface and impression tags. Arrows indicate impression materials; Asterisks show examples of the impression of dentinal tubules.

Twenty-seven samples were analyzed by SEM to determine the penetration of the dental materials into the dentinal tubules. No differences in terms of material remnants between the impression materials and surfaces of the teeth were evident to the naked eye. Under the scanning electron microscopy investigation, material tags and material remnants were observed for all of the three of the experimental groups, as shown in Figure 2. After the Euclidean analysis, the mean lengths, diameters and fractal analysis (*Df)* (±SD) of the impression tags were reported in Table 1. These data showed that impression tags were present for all of the experimental groups, thus rejecting the H0 hypothesis. Hypothesis H1 was defined in terms of the presence of the impression material, which was also evaluated through statistical Bayesian ANOVA analysis. In terms of presence of impression tags, a statistically significant difference was shown between the experimental (polyether and polyvinyl siloxane) groups.

**Figure 3:**
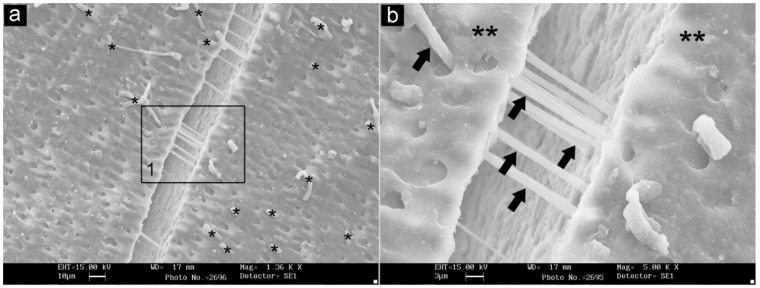
(**a,b**) Detail of a sample broken open to see the impression materials inside the dentinal tubules. Asterisks and arrows (b) show examples of the materials trapped in the dentinal tubules.

Figure 3 illustrates details of a sample that was broken and opened to see the impression materials inside the tubules. Moreover, the statistical analysis performed between the groups showed significant differences for the tag lengths between the two experimental groups and teeth (*p* < 0.0001). A statistically significant difference was also shown in terms of tags diameters between the impression materials and teeth (*p* < 0.0001), whilst no statistically significant difference was reported between the polyether and polyvinylsiloxane. In addition, the analysis of the fractal dimension reported significant difference between the experimental groups when compared to natural teeth (*p* < 0.0001). Details of the comparison between the groups taken into consideration are shown in Table 2 which demonstrates that the impression materials can invade the spaces in the newly cut dentinal tubules under all of the experimental conditions.

## 4. Discussion

The null hypothesis in this study was rejected as the impression material was found inside the dentinal tubules, and the impression tags were present on the impression material. Therefore, the alternative H1 hypothesis that rejected the H0 hypothesis was evaluated. The alternative hypothesis demonstrated that the presence of the tags was statistically significant in the samples under examination, and therefore relevant from the clinical point of view. It is now known that the interface area between the impression material and the tooth is the most complex to manage due to the potential for elastic deformation during removal [38]. For this reason, the characteristics of the elastic deformation during the phase of removal of the impression are among the most studied rheological characteristics of impression materials [39,40]. In the same way, another key feature is the tensile strength of a material, which is defined as “the ability of a material to resist tearing in thin areas” [41]. In this sense, the data presented herein demonstrate for the first time not only that impression materials can penetrate into the dentinal tubules but also that this phenomenon was essentially superimposable for the two impression materials in this study. This demonstrated how both of these impression materials have high intrinsic characteristics and provided a high degree of accuracy.

In terms of the global characteristics, previous studies have shown that the polyether and PVS impression materials compared here have good mechanical properties and show acceptable levels of distortion [42,43]. Indeed, a study that compared polyether and PVS showed no significant differences under dry conditions in terms of the dimensional distortion [43]. In the present study, the lack of significant differences between these two types of elastomeric impression materials under this testing suggested that both of these materials can reproduce the required details. Moreover, the diameters of the tags were not significantly different, which reinforces the hypothesis of impression material penetration along the tubules.

On the other hand, in the comparison of the tags for the impression materials on the dentine with those for the control group (i.e., the tooth samples), significant differences were seen. The differences between the lengths of the polyether filaments can be attributed to the contraction of the impression material tags after breaking inside the tubules. The material that penetrated into the tubules became detached, showing the presence of residual material inside the tubules; however, shorter lengths for the tooth surface compared with the “in positive” tags of the impression material. These data therefore show contamination of the freshly cut dentinal tubules when the impression material was applied to the surface.

To the best of our knowledge, although contamination of dentinal tubules with different impression agents is an accepted phenomenon, there have been no previous studies for the definition of this contamination within the dentinal tubules [25,27,28,29,30]. Many studies have shown that to have a good adhesion between resin and tooth, and consequently for a good restoration, it was fundamental that the adhesive substrate should not be contaminated. Agents such as blood, gingival fluid, haemostatic fluid, saliva and lubricant oils can negatively affect the duration of restorations, which can be associated with caries, detachment, sensitivity and pigmentation of the substrate [27,28,29,30]. Therefore, the results of the present study showed how even the impression material used immediately after dental preparation can contaminate the dentine substrate. This might affect the adhesive properties of the adhesives and resins. For this reason, a different approach that foresees the sealing of the tubules immediately after the preparation should provide positive benefits, as already indicated in the literature [17,18,19,22].

Some studies have shown that the use of the IDS techniques can preserve the freshly cut dentinal tubules. In the same way, however, it was hypothesized that the same impression material can interact negatively on the adhesive layer applied before the impression, as recommended in the IDS protocol [19,22,23]. In a recent study in 2019, Sinjari et al. [23] demonstrated how an adequate surface cleaning protocol can reset the interactions with the impression material. In their study, they proposed that following the adhesion protocol, the surface should be cleaned with prophy paste and a surfactant agent (e.g., Marseille soap). They thus used SEM analysis to demonstrate how this procedure can reset the residues of the impression materials present on the teeth, to ensure total polymerization of the material itself, without reacting with the surface layer of the adhesive. Therefore, together with the data shown herein, the interactions of the impression materials are a key phenomenon following dental preparation. Freshly cut dentine can trap residues of impression materials inside. This phenomenon should be avoided to reduce the risk of contamination of the tubules. The IDS protocols proposed in the literature were further supported by these data, which provided essential information on the interactions of the materials with freshly cut teeth.

The major limitation of the present study was related to the absence of dentinal fluid and pulp pressure. Although, the in vivo pulp pressure has been reported as 14.1 cm of H_2_O [44], this might still be too low to avoid the impression material penetration into the dentinal tubules. More studies with simulated pulp pressure are needed to clarify the full significance of this phenomenon.

## 5. Conclusions

In conclusion, the data in the present in vitro investigation have shown that the impression materials can penetrate and remain entrapped inside the dentinal tubules during the impression procedures. This aspect might have a negative influence on the adhesion, and thus this study strongly supports the use of an immediate dentine sealing procedure for adhesive restorations.

## Figures and Tables

**Table 1:** Descriptive statistics of the Euclidean and fractal dimension analyses.

Descriptive	Length			Diameter			Fractal Dimension		
Statistic	Polyether	Polyvinyl Siloxane	Tooth	Polyether	Polyvinyl Siloxane	Tooth	Polyether	Polyvinyl Siloxane	Tooth
Measurements (n)	58	65	37	58	65	37	58	65	37
Missing (n)	0	0	0	0	0	0	0	0	0
Mean (µm)	22.60	21.89	11.32	2.84	2.46	3.12	1.78	1.77	1.71
Std. deviation (µm)	11.05	12.85	7.00	0.51	0.75	0.72	0.03	0.03	0.03

**Table 2:** Analysis for the presence of impression tags between the groups in terms of length, diameter and fractal dimension. p < 0.05 (ANOVA; with post hoc Tukey’s multiple comparison tests).

Comparator	Comparison	Tukey’s Multiple Comparisons Test		
		Mean Difference	95% Confidence Interval of Difference	Significance
Length (µm)	Polyether vs. Polyvinyl siloxane	0.71	−4.03 to 5.45	*p* = 0.8818
	Polyether vs. Teeth	11.27	5.74 to 16.79	*p* < 0.0001
	Polyvinyl siloxane vs. Teeth	10.56	5.15 to 15.97	*p* < 0.0001
Diameter (µm)	Polyether vs. Polyvinyl siloxane	0.38	0.09 to 0.66	*p* = 0.01
	Polyether vs. Teeth	−0.28	−0.61 to 0.05	*p* = 0.12
	Polyvinyl siloxane vs. Teeth	−0.66	−0.98 to −0.03	*p* < 0.0001
Fractal dimension	Polyether vs. Polyvinyl siloxane	0.01	−0.00 to 0.02	*p* = 0.24
	Polyether vs. Teeth	0.07	0.05 to 0.08	*p* < 0.0001
	Polyvinyl siloxane vs. Teeth	0.06	0.04 to 0.07	*p* < 0.0001
